# Retraction Note: Culture of previously uncultured members of the human gut microbiota by culturomics

**DOI:** 10.1038/s41564-024-01894-5

**Published:** 2024-11-22

**Authors:** Jean-Christophe Lagier, Saber Khelaifia, Maryam Tidjani Alou, Sokhna Ndongo, Niokhor Dione, Perrine Hugon, Aurelia Caputo, Frédéric Cadoret, Sory Ibrahima Traore, El Hadji Seck, Gregory Dubourg, Guillaume Durand, Gaël Mourembou, Elodie Guilhot, Amadou Togo, Sara Bellali, Dipankar Bachar, Nadim Cassir, Fadi Bittar, Jérémy Delerce, Morgane Mailhe, Davide Ricaboni, Melhem Bilen, Nicole Prisca Makaya Dangui Nieko, Ndeye Mery Dia Badiane, Camille Valles, Donia Mouelhi, Khoudia Diop, Matthieu Million, Didier Musso, Jônatas Abrahão, Esam Ibraheem Azhar, Fehmida Bibi, Muhammad Yasir, Aldiouma Diallo, Cheikh Sokhna, Felix Djossou, Véronique Vitton, Catherine Robert, Jean Marc Rolain, Bernard La Scola, Pierre-Edouard Fournier, Anthony Levasseur, Didier Raoult

**Affiliations:** 1 Aix Marseille Université URMITE, UM63, CNRS 7278, IRD 198, INSERM 1095, 27 Boulevard Jean Moulin, Marseille, Cedex 5 France; 2 Institut Louis Malardé, Papeete, Tahiti, Polynésie Française; 3 Departamento de Microbiologia Laboratorio de Virus, Universidade Federal de Minas Gerais, Belo Horizonte, Brasil; 4 Special Infectious Agents Unit, King Fahd Medical Research Center, King Abdulaziz University, Jeddah, Saudi Arabia; 5 Institut de Recherche pour le Développement, UMR 198 (URMITE), Campus International de Hann, IRD, BP 1386, CP, Dakar, Sénégal; 6 Department of Infectious and Tropical Diseases, Centre Hospitalier de Cayenne, Cayenne, French Guiana; 7 Service de Gastroentérologie, Hôpital Nord, Assistance Publique-Hôpitaux de Marseille, Marseille, France

Retraction to: *Nature Microbiology* 10.1038/nmicrobiol.2016.203, published online 7 November 2016.

The Editors have retracted this article. The article cites approval from an institutional ethics committee in France, but it also reports the analysis of samples collected from individuals in several other countries. The authors were not able to provide documentation of approval from appropriate ethics committees in each of these additional countries, or of compliance with local regulations regarding the use of such samples in research.

Jônatas Abrahão agrees with this retraction. Jean-Christophe Lagier, Matthieu Million, Bernard La Scola, Gregory Dubourg, Maryam Tidjani Alou, Gaël Mourembou, Sory Ibrahima Traore, Cheikh Sokhna, Camille Valles, Anthony Levasseur, Pierre-Edouard Fournier, Jean Marc Rolain, Sara Bellali, Elodie Guilhot, Ndeye Mery Dia Badiane, Aurelia Caputo, Saber Khelaifia, Esam Ibraheem Azhar, Fehmida Bibi, and Muhammad Yasir disagree with this retraction. Didier Raoult did not explicitly state whether they agree or disagree with this retraction. The remaining authors did not respond to correspondence from the Publisher about this retraction.

